# Randomized trial of surveillance with abbreviated MRI in women with a personal history of breast cancer– impact on patient anxiety and cancer detection

**DOI:** 10.1186/s12885-022-09792-x

**Published:** 2022-07-15

**Authors:** Marina Mohallem Fonseca, Tasneen Alhassan, Yashmin Nisha, Diana Koszycki, Betty Anne Schwarz, Roanne Segal, Angel Arnaout, Tim Ramsay, Jacqueline Lau, Jean M. Seely

**Affiliations:** 1 Breast Imaging fellow 2017-2018, Former University of Ottawa, Now Annecy, France; 2 Breast Imaging fellow 2016-2017, Former University of Ottawa, Now Dubai, United Arab Emirates; 3 University of Ottawa, Breast Imaging fellow, Ottawa, 2019-2020 Canada; 4 Research Chair in Mental Health, Institut du Savoir Montfort, Ottawa, Canada; 5 Faculty of Education (Counselling Psychology), Faculty of Medicine (Psychiatry), Institut du Savoir Monfort, Ottawa, Canada; 6 Department of Medical Imaging, The Ottawa Hospital, Ottawa, Canada; 7 Department of Medicine, Oncology, The Ottawa Hospital Cancer Center, University of Ottawa, Ottawa, Canada; 8 Breast Surgical Oncology and Oncoplastic Surgery, University of Ottawa, Ottawa Hospital Research Institute, Ottawa, Canada; 9 Clinical Epidemiology Program, School of Epidemiology and Public Health, Ottawa Hospital Research Institute, University of Ottawa, Ottawa, Canada; 10 Department of Radiology, University of Ottawa, Ottawa, Canada; 11 Departments of Radiology and Surgery, Department of Medical Imaging, The Ottawa Hospital, Ottawa Hospital Research Institute, University of Ottawa, General Campus, 501 Smyth Rd, Ottawa, ON K1H 8L6 Canada

**Keywords:** Breast cancer, MRI, Anxiety, Abbreviated, Mammography

## Abstract

**Background:**

Abbreviated breast MRI (A-MRI) substantially reduces the image acquisition and reading times and has been reported to have similar diagnostic accuracy as a full diagnostic protocol but has not been evaluated prospectively with respect to impact on psychological distress in women with a prior history of breast cancer (PHBC). This study aimed to determine if surveillance mammography (MG) plus A-MRI reduced psychological distress and if A-MRI improved cancer detection rates (CDR) as compared to MG alone.

**Methods:**

This prospective controlled trial of parallel design was performed at a tertiary cancer center on asymptomatic women with PHBC who were randomized into two groups: routine surveillance with MG or intervention of MG plus A-MRI in a 1:1 ratio. Primary outcome was anxiety measured by four validated questionnaires at three different time-points during the study. Other parameters including CDR and positive predictive value for biopsy (PPV3) were compared between imaging modalities of MG and A-MRI. Tissue diagnoses or 1 year of follow-up were used to establish the reference standard. Linear mixed models were used to analyze anxiety measures and Fisher’s exact test to compare imaging outcomes.

**Results:**

One hundred ninety-eight patients were allocated to either MG alone (94) or MG plus A-MRI (104). No significant group difference emerged for improvement in trait anxiety, worry and perceived health status (all Time-by-surveillance group interaction ps > .05). There was some advantage of A-MRI in reducing state anxiety at Time 2 (*p* < .05). Anxiety scores in all questionnaires were similarly elevated in both groups (50.99 ± 4.6 with MG alone vs 51.73 ± 2.56 with MG plus A-MRI, *p* > 0.05) and did not change over time. A-MRI detected 5 invasive cancers and 1 ductal carcinoma in situ (DCIS), and MG detected 1 DCIS. A-MRI had higher incremental CDR (48/1000(5/104) vs MG 5/1000(1/198, *p* = 0.01)) and higher biopsy rates (19.2% (20/104) vs MG 2.1% (2/94), *p* < 0.00001) with no difference in PPV3 (A-MRI 28.6% (6/21) vs MG 16.7% (1/6, *p* > .05).

**Conclusion:**

There was no significant impact of A-MRI to patient anxiety or perceived health status. Compared to MG alone, A-MRI had significantly higher incremental cancer detection in PHBC. Despite a higher rate of biopsies, A-MRI had no demonstrable impact on anxiety, worry, and perceived health status.

**Trial registration:**

ClinicalTrials.gov (NCT02244593). Prospectively registered on Sept. 14, 2014.

**Supplementary Information:**

The online version contains supplementary material available at 10.1186/s12885-022-09792-x.

## Background

Women with a prior personal history of breast cancer (PHBC) often have a high level of anxiety related to breast cancer surveillance [1]. Their actual recurrence rates are estimated in the order of 1% per year [2, 3], and depend on tumor size, histology and nodal status at diagnosis, with 5-year risks of recurrence of 7% for stage I, 11% for Stage II and 13% for stage III, and distant recurrences of 10- 41% at 20 years after completion of adjuvant chemotherapy [4, 5]. Early detection decreases mortality for women with breast cancer [6–8]. In women with PHBC, the survival benefit is improved if new or recurrent breast cancer is found on surveillance mammography (MG) instead of physical examination [9]. However, MG has been shown to be less sensitive in women with PHBC, with sensitivity of 65.4% compared with 76.5% in women with no PHBC [10]. Breast MRI is the most sensitive test for detecting breast cancer [11]. Breast MRI is currently recommended for women with PHBC and dense tissue or those diagnosed by age 50, as per American College of Radiology (ACR) guidelines [12]. Several other national guidelines do not recommend surveillance imaging with breast MRI after a personal history of breast cancer unless someone has a hereditary mutation or mammographically occult malignancy and of itself, breast tissue density is not an indication for surveillance breast MRI. Compliance with MRI screening has been shown to be low, on the order of 25%, due both to lack of availability and high costs associated with lengthy acquisition times [13, 14]. Abbreviated breast MRI (A-MRI), which substantially reduces the image acquisition and reading times, has been reported to have similar diagnostic accuracy as a full diagnostic protocol [13, 15–19]. Currently, A-MRI has not been adopted as the standard for screening for breast cancer and more studies are required to evaluate outcomes.

Prior studies demonstrated that supplementary MRI surveillance in women at high risk of breast cancer does not impact anxiety, cancer-specific distress or health-related quality of life [1, 20]. This is the first study to our knowledge to evaluate the psychological effect of adding abbreviated MRI to MG surveillance in women with PHBC.

The primary purpose of the current study was to determine if the intervention of adding A-MRI to MG surveillance was more effective than MG alone in reducing patient anxiety and, secondarily, if A-MRI improved cancer detection in women with PHBC. Our hypothesis was that the MRI group would be superior to mammography alone group to reduce patient anxiety.

## Methods

### Study subjects

This prospective randomized controlled trial of parallel design was performed at a large tertiary care academic medical center and was approved by the hospital’s institutional review board. Our study adheres to the CONSORT guidelines. Patients at a single tertiary care cancer center were approached by their treating oncologists or surgeons during routine clinical appointments if they met the eligibility criteria and their scheduled appointment time allowed. The patients’ oncologists or surgeons obtained written informed consent. Eight oncologists and three breast surgeons recruited patients between 2/1/2015 and 4/30/2019. Patients were followed for a minimum of 12 months.

The eligibility criteria included: (a) female patients 18 years or older; (b) PHBC (including DCIS and invasive ductal or lobular carcinoma); (c) prior unilateral mastectomy or breast conservation surgery; (d) treatment for breast cancer completed; and (e) no symptoms of breast cancer. Patients were excluded if they were considered high-risk (lifetime risk ≥ 25%) [21], were unable to undergo an MRI due to either physical or mental issues (i.e.: severe claustrophobia, allergy to gadolinium, severe renal failure), had bilateral mastectomies, were pregnant or breastfeeding, or had undergone a breast MRI within the last 6 months. Regular surveillance imaging consisted of annual surveillance MG, irrespective of breast tissue density. All patients had undergone prior mammographic imaging, and some (< 50%) had undergone prior breast MRI imaging.

Eligible patients were randomized in a 1:1 allocation ratio to one of the two arms of the study: 1) surveillance with MG or 2) MG plus A-MRI, with use of permuted blocks of variable length (2, 4, and 6) to ensure that recruiting physicians remained unaware of the randomization. Researchers or study participants were not blinded to their allocation. Patients could only participate once in the study.

### Imaging technique and interpretation

All mammographic examinations were performed using a full-field digital technique (Hologic, Bedford, MA, USA) in accordance with national guidelines. Standard two-dimensional craniocaudal (CC) and mediolateral oblique (MLO) views were obtained.

All abbreviated dynamic contrast material-enhanced breast MRIs were performed with one 3 T system (Magnetom TrioTim Syngo, Siemens). The standardized protocol consisted of 8-channel breast coil (Sentinelle Medical Inc.), T1 localizer, T1 dynamic contrast-enhanced fat-suppressed with one precontrast and one 2 min postcontrast (3D transverse, phase encoding direction right to left, phase resolution of 60%, phase partial Fourier 6/8, no interpolation, FA 10 degrees, TR 4.07 ms and TE 1.96 ms, no IR, NEX 1, Voxel size: 1 × 1x1 mm, acceleration factor 4, no interpolation, base resolution 448,1:01 min, slice thickness 1 mm). Post-processing axial subtracted sequences and axial and sagittal maximum intensity projection were generated of the subtracted images. No T2-weighted sequences were obtained. For all examinations, gadolinium contrast material (Gadovist) was power injected (0.1 mmol/kg at 2 mL/s) followed by a 20 mL saline flush. The entire protocol took 3 min.

Surveillance MG and A-MRI were reviewed by one of two breast radiologists independently (the first with 8 years of experience reading mammography and breast MRI and the second reader with 20 years reading mammography and breast MRI) using ACR Breast Imaging-Reporting Data System (BI-RADS) lexicon[22]. For patients in the A-MRI group, MG and A-MRI studies were performed on the same day according to the protocol. Radiologists were not blinded but reported each modality separately according to the imaging modality findings, with the mammograms interpreted first. Based on the imaging findings, additional mammographic images, including diagnostic tomosynthesis, or targeted ultrasound were requested at the discretion of the interpreting radiologist. Findings and management were communicated to the patient by telephone by the reporting radiologist. Subsequent imaging was performed on separate visits, within 3 weeks of the MG or A-MRI. Histologic samples for pathologic diagnosis were obtained under ultrasound (14G, 5–6 cores), stereotactic (10G, 6–12 cores) or MRI (10G, 6–12 cores) guidance.

### Anxiety measures

Patients in both groups were asked to fill out four validated self-report questionnaires that measure anxiety level and overall health [23–26]) (see supplemental materials). The primary outcome was the State-Trait Anxiety Inventory (STAI) [23]. This STAI consists of two separate 20-item scales that assess state anxiety (S-Anxiety) (i.e., how the person feels at this moment) and trait anxiety (T-Anxiety) (i.e., how the personal generally feels). The items are rated on a 1 to 4 scale with total scores ranging from 20–80. Cut-off scores of ≥ 32.2 and ≥ 31.8 indicate elevated levels of state and trait anxiety, respectively. Both STAI scales have solid psychometric properties and are sensitive to assessment of longitudinal change. There are no validated cutoff scores for the STAI scales in women with PHBC, however a cutoff score of 41 on the trait form of the STAI and 44 on the state form of the STAI have been used in previous research to identify clinical levels of anxiety in women with breast cancer [27, 28]. Other psychological measures included the Penn State Worry Questionnaire (PSWQ) [24], Breast Cancer Worry Scale (BCWS) [25], and the Health Status Questionnaire 12 (HSQ-12) [26]. The PSWQ [24] is a 16-item self-report questionnaire which measures frequency and intensity of worry symptoms. Items are rated on a 5-point scale, with total scores ranging from 16–80. A score between 16–39 indicates low worry, 40–59 moderate worry and 60–80 high worry. The BCWS [25] is a 3-item scale which measures frequency of breast cancer worry and the impact of worrying on mood and ability to perform daily activities. Higher scores indicate greater cancer worry. The HSQ-12 [26] assesses the impact of health on social, emotional and physical functioning over the past four weeks. Depending on the item, questions are rated of a 3-point, 5-point and 6-point scale. Items were recoded using the method described by Barry et al. [26]. Total HSQ scores range from 0 to 800, with higher scores indicating better health status. The questionnaires were completed upon enrolment during consultation at time 1 (T1) when the patients were due for their surveillance test(s) to measure baseline levels of anxiety, at time 2 (T2) that occurred after the patient received of their surveillance MG and/or MRI test results, and then 6 months later at time 3 (T3), to determine if there was a sustained effect observed from the type of surveillance test. T3 questionnaires were mailed to patients and returned to the study coordination center.

### Data collection and statistical analysis

Medical records were reviewed to determine patient age, family history of breast and/or ovarian cancer in a first-degree relative, surgery modality, initial breast tumor stage (TNM), histology, hormone receptor status, months since diagnosis of breast cancer and breast density. Results were compared between the two groups. For malignant or atypical/high-risk lesions, surgical pathologic results were reviewed when available. Imaging and clinical follow-up were determined by review of the hospital picture archiving system (PACS) and medical records as well as the digital imaging repository which includes all clinics and hospitals that serve the region’s population of 1.2 million. The emigration rate in the region is < 0.5% per year [29]. Imaging follow-up for all patients with benign imaging or pathology was documented with the date of the most recent negative mammogram.

The anxiety measures were analyzed using SPSS Statistics version 25. Analysis was based on intent-to-treat (ITT) principles. Data were analyzed using linear mixed models, with surveillance groups (MG only versus MG plus A-MRI), time of assessment (T1, T2, T3), and Intervention by Time interaction as fixed factors. Models were estimated using Restricted Maximum Likelihood (REML) with an unstructured covariance structure to account for correlations among repeated measures over time. A significant Time by surveillance group interaction would suggest that changes in measures over time were different between the surveillance method; significant interactions were further analyzed with pairwise least square mean comparisons. Data from missing questionnaires were not imputed because our analytical strategy using REML allowed the estimation of reliable parameters without the need for imputation of the data under an assumption of missing at random (MAR) [30]. Descriptive statistics were calculated using a spreadsheet software program (Excel, Version 2013, Microsoft). Screening outcomes were compared between groups using Fisher’s exact test. Sample size calculation was based on primary outcome the STAI. There is no generally accepted minimal clinically important difference in the STAI subscales and a 4-point difference was selected to be a minimal clinically important difference. This was based on previous study by Millar et al. [28] which used a 4-point difference in the STAI and on consensus with the research team and the experience of the psychologist researcher. In order to have 80% power to detect a 4-point difference between the groups at any of the three time points, we planned 134 patients per group. Recruitment stopped early due to differences in cancer detection rates (CDR). Results were considered significant if *p* < 0.05.

Imaging modalities (MG, A-MRI), and BI-RADS final assessment categories for each modality were noted. Imaging findings and outcomes were documented for all BI-RADS 3, 4 and 5 lesions, including suspicious extra-mammary findings. Results were compared between MG and A-MRI. A screening examination was considered as positive when additional diagnostic imaging was recommended prior to the next routine screening examination and included BI-RADS 0, 3, 4 and 5, defined as abnormal interpretations. True positive findings were defined as a cancer diagnosis within 12 months of a positive screening examination. Imaging studies were considered false negatives if there was a tissue diagnosis of cancer within 12 months of a negative study, or in the surveillance groups if there was a tissue diagnosis of cancer in the follow-up period. The following performance metrics were calculated for each modality: CDR, abnormal interpretation rate (AIR), biopsy rate, positive predictive value for biopsy recommendations (PPV2 = biopsies recommended/cancers diagnosed), positive predictive value for biopsies performed (PPV3 = biopsies performed/cancers diagnosed), sensitivity and specificity.

## Results

**Fig. 1:**
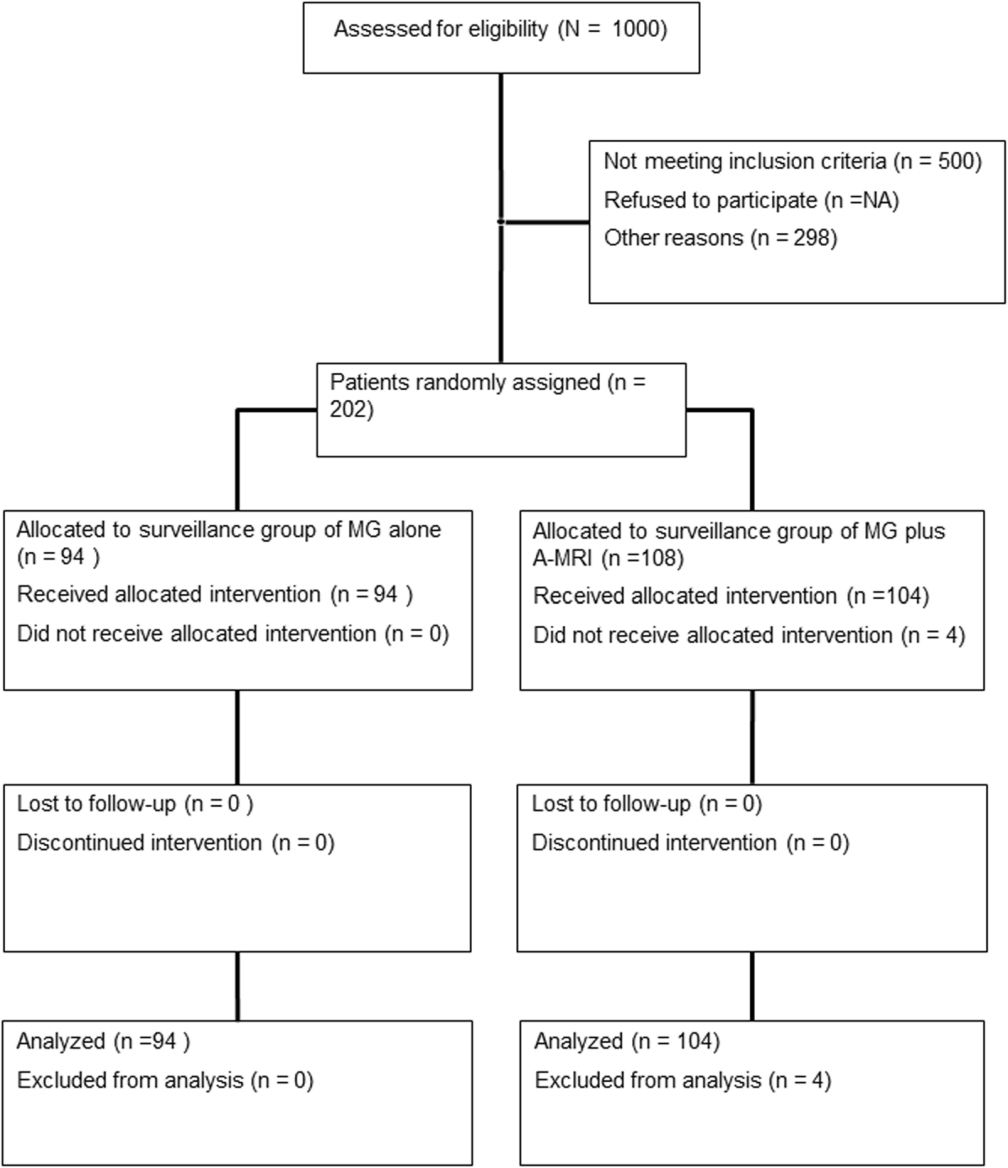
CONSORT Flow diagram of trial

A total of 202 of 1000 patients fulfilled the eligibility criteria (Fig. 1) between 2/1/2015 and 4/30/2019. At enrollment, 94 were randomized to surveillance with MG alone and 108 to MG plus A-MRI. Of these, four patients from MG plus A-MRI group withdrew from the study a few days before undergoing the imaging for different reasons: two patients were discovered to have breast cancer metastases on separate imaging done prior to undergoing the surveillance imaging, one patient developed sepsis before the imaging was performed and her doctor decided to postpone contrast injection and one patient opted to withdraw from the study before undergoing the imaging. Accordingly, the study population consisted of 198 patients: 47.5% (94/198) randomized to regular surveillance with MG and 52.5% (104/198) to surveillance with MG plus A-MRI. All patients completed the imaging to which they were randomized and there were no patient crossovers from the MG only group to A-MRI. Among the 104 patients who had MG plus A-MRI, 82.7% (86/104) had both imaging exams the same day and 17.3% (18/104) on different days (average 33.2 days (range: 1–147)) due to various scheduling conflicts.

### Baseline demographic and clinical characteristics

Patients’ demographic and clinical characteristics are presented in Table 1. No important differences in age, family history of breast and/or ovarian cancer, surgery modality, months since diagnosis, breast density, initial tumor histology, stage, or hormone receptor status were noted between the two groups, although a nonsignificant higher number of patient with triple negative cancers was observed in the group that received A-MRI.

**Table 1:** Patient demographics according to group (MG *versus* A-MRI + MG)

	**Group 1-MG (*n* = 94)**	**%**	**Group 2-A-MRI + MG (*n* = 104)**	**%**	***P* value**^b^
Age (years)					
Mean	59.0		58.2		0.44
Median	58.5		58		
Range	35–80		38–83		
Family history breast and/ or ovarian cancer					
Yes	26	28%	32	31%	0.63
No	68	72%	71	68%	0.53
Unknown	0	0%	1	1%	0.34
Surgery modality					
Lumpectomy	52	55%	69	66%	0.11
Mastectomy	42	45%	35^a^	34%	0.11
Months since diagnosis					
< 24	42	45%	44	42%	0.73
24 ≤ x < 60	37	39%	39	38%	0.79
60 ≤ x ≤ 120	13	14%	14	13%	0.94
> 120	2	2%	6	6%	0.19
Unknown	0	0%	1	1%	0.34
Breast density					
ACR A	4	4%	7	7%	0.45
ACR B	45	48%	46	44%	0.61
ACR C	44	47%	45	43%	0.50
ACR D	1	1%	6	6%	0.73
Tumor histology					
Ductal carcinoma in situ	2	2%	7	7%	0.12
Invasive ductal carcinoma	84	89%	89	86%	0.31
Invasive lobular carcinoma	6	6%	8	8%	0.74
Mucinous carcinoma	1	1%	0	0%	NA
Unknown	1	1%	0	0%	NA
Tumor stage of initial invasive cancer					
T1	51	54%	44	42%	0.19
T2	27	29%	38	37%	0.14
T3	5	5%	8	8%	0.43
T4	8	9%	5	5%	0.35
NA	1	1%	2	2%	0.59
N0,NX	51	54%	54	52%	0.92
N1	31	33%	33	32%	0.92
M0	94	100%	104	100%	NA
Hormone receptor status invasive cancer					
ER positive Her2 negative	69	73%	66	63%	0.33
Her2 positive	12	13%	19	18%	0.21
Triple negative	6	6%	12	12%	0.16
Unknown	4	4%	0	0%	NA

^a^two patients had mastectomy and contralateral lumpectomy

^b^using Fisher’s exact test for comparison between the two groups

### Results regarding anxiety

The observed means (± standard deviations) for the self-report questionnaires and least square mean difference between the surveillance groups at T2 and T3 are displayed in Table 2. 197 participants completed questionnaires at T1 (Baseline), 92 patients in MG only and 105 in MG plus A-MRI groups. At Time 2, data were available for 143 participants; 60 in MG and 83 in MG plus A-MRI groups. At T3 data were available for 102 participants; 38 women in MG and 64 in MG plus A-MRI groups. The surveillance groups did not differ significantly on any of the baseline measures. Linear mixed models revealed that our primary outcome STAI-Trait Anxiety did not change over time (Time main effect *p* = 0.51) and did not differ between the groups (Time x Surveillance Group interaction *p* = 0.20). However, there was a significant Time main effect (*p* < 0.001) and Time x Surveillance Group interaction (*p* = 0.022) for the STAI-State Anxiety. Post-hoc tests revealed that for both groups, state anxiety decreased significantly between T1 and T2 (estimated mean change = -6.80 [95% CI, -8.58 to -5.02] for MG only (*p* < 0.001) and -8.17 [95% *CI*, -9.70 to -6.34] for MG plus A-MR (*p* < 0.001), and increased significantly from T2 to T3 (estimated mean change 5.86 [95% *CI*, 3.97 to 7.76] for MG only (*p* < 0.001) and 8.12 [95% *CI,* 7.23 to 10.40] for MG plus A-MRI (*p* < 0.001). Between groups comparisons indicated that state anxiety at T2 was significantly lower in the MG plus A-MRI group (*p* = 0.03), but less than a 4-point difference. Levels of worry did not significantly change over time (Time main effect *p* = 0.14 and *p* = 0.73 for the PSWQ and BCWQ, respectively) and did not differ between the groups (Time x Surveillance Group interaction *p* = 0.57 and *p* = 0.48 for the PSWQ and BCWQ, respectively). There was a significant Time main effect for self-report health status (*p* < 0.05), but no significant Time x Surveillance Group interaction (*p* = 0.13). Overall, HSQ-12 scores decreased from T1 to T3 (estimated mean change -0.27.91 [95% CI -47.60 to -8.22], *p* < 01).

**Table 2:** Effect of Surveillance Method on Self-Report Measures

	**Observed means ± standard deviations**				
**Outcome**	**Time 1**	**Time 2**	**Time 3**	**Estimated mean difference between groups at Time 2 (95% CI)**	**Estimated mean difference between groups at Time 3 (95% CI)**
**STAI-Trait**					
MG alone	51.50 ± 2.6	51.55 ± 2.8	51.79 ± 2.5	-0.37 (-1.23 to 0.49)	-0.80 (-1.71 to 0.10)
MG + A- MRI	51.61 ± 2.5	51.20 ± 2.2	50.94 ± 2.5		
**STAI-State**					
MG alone	51.85 ± 2.8	45.14 ± 5.7	50.99 ± 4.6	-2.16 (-4.20 to 0.13)*	- 0.79 (-0.48 to 2.07)
MG + A-MRI	51.07 ± 2.9	42.88 ± 6.3	51.73 ± 2.6		
**PSWQ**					
MG alone	40.23 ± 13.7	39.52 ± 13.4	38.98 ± 14.2	0.83 (-3.4 to 5.1)	2.43 (-1.98 to 6.84)
MG + A-MRI	42.20 ± 13.4	41.08 ± 14.5	44.41 ± 14.2		
**BCWS**					
MG alone	6.87 ± 2.4	6.93 ± 2.4	6.44 ± 2.1	-0.40 (-1.11 to 0.30)	-0.27 (-0.99 to 0.45)
MG + A-MRI	6.73 ± 2.3	6.55 ± 2.3	6.39 ± 2.1		
**HSQ-12**					
MG alone	617.52 ± 143.7	603.57 ± 122.5	590.54 ± 156.3	-6.12 (-48.48 to 36.34)	25.41 (-23.32 to 74.13)
MG + A-MRI	597.94 ± 145.3	596.73 ± 141.5	611.44 ± 123.5		

*Note*: Analysis is based on the intent-to-treat sample. Questionnaires were completed upon enrolment during consultation (T1; *n* = 92 MG alone and *n* = 103 MG + A-MRI), upon receipt of the MG and/or MRI results (T2; *n* = 61 MG alone and *n* = 84 MG + A-MRI), and 6- months later (T3; *n* = 49 MG alone *n* = 73 MG + A-MRI)

For all questionnaires, higher scores represent higher anxiety or worry

*STAI* State Trait Anxiety Trait (i.e., how the personal generally feels) and State (i.e., how the person feels at this moment), *PSWQ* Penn State Worry Questionnaire, *BCWQ* Breast Cancer Worry Scale, *HSQ* Health Status Questionnaire

Range of scores: For the trait-STAI scores range from 20–80 and the state-STAI scores range from 20–80, with cut-off scores of ≥ 31.8 and ≥ 32.2 indicating elevated levels of trait and state anxiety, respectively. The PSWQ scores range from 16–80 (higher scores indicating higher worry), and the BCWS summed scores range from 3 (low worry) to 12 (high worry). The scores for the HSQ-12 range from 0 to 800, with higher scores indicating better perceived health status

^*^p < *.05 MG alone vs MG* + *A- MRI*

Using the cutoff score of 41 on the trait form of the STAI, the percentage of women with anxiety in the clinical range was 100% at T1 and T2 and T3 for both the MG and the MG + A-MRI groups. Using a cutoff score of 44 on the state form of the STAI, the majority of women in the MG and MG + A-MRI groups had scores in the clinical range at T1 (100% and 99%) and T3 (95.9% and 100%). At T2 however, more (57.1% (32/56)) women in the MG group than those in MG + A-MRI group (32.9% (27/82)) had scores in the clinical range, with the difference between groups statistically significant (*p* < 0.01). Within the MRI group, the 23 recalled patients had significantly higher PSWQ scores at T2 when compared with the 80 patients who were not recalled (mean score: 47.16 ± 14.7 vs 39.33 ± 14.08, *p* < 0.05, Cohen’s d = 0.55), with a similar trend found for State STAI scores (45.22 ± 7.1 vs 42.23 ± 5.95, *p* = 0.075, Cohen’s d = 0.48). However, the recalled women had scores that returned to baseline by T3 with no lasting effect on PSWQ (mean score: 48.66 ± 16.29 vs 43.30 ± 13.51, *p* > 0.05) or State STAI (52.20 ± 2.32 vs 51.58 ± 2.63, *p* > 0.05).

### Outcomes according to surveillance groups

There was 1 cancer (DCIS) detected in the MG group during the study, and 3 cancers (2 invasive and 1 DCIS) were diagnosed at follow-up. In the MG plus A-MRI surveillance group, 5 breast cancers (1 DCIS and 4 invasive cancers) and 1 breast cancer metastasis to the lung were detected, with no cancers diagnosed at follow-up. The outcomes for each group are provided in Table 3. Although there were significantly more recalls and biopsies performed in the MG plus A-MRI than the MG group (recalls of 27/104 (26%) vs 4/94 (4.26%) (*p* < 0.05) and biopsies of 20/104 (19.2%) vs 2/94 (2.13%)(*p* = 0.001) respectively), with lower specificity (77.8% MG + A-MRI vs 96.7% MG (*p* = 0.0001), the sensitivity for the MG plus A-MRI group was higher 5/5(100%) than the MG group 1/4 (25%)(*p* = 0.048) and there was a higher CDR in the MG plus A-MRI group (5/104 (48.1/1000)) than the MG group (1/94 (10.6/1000) (*p* = 0.1294)).

**Table 3:** Outcomes according to surveillance group

	**MG group *n* = 94**	**MG + A-MRI group *n* = 104, breast findings only**	**MG + A-MRI group *n* = 104, including extramammary findings**	
**Outcome**				*P* value**
False negative^a^	3	0	0	-
True negative	87	77	74	-
False positive^b^	3	22	24	-
True positive	1	5	6	-
Recall rate	4.3% (4/94)	26% (27/104)	28.9% (30/104)	< 0.0001
Biopsy rate	2.1% (2/94)	19.2% (20/104)	21.2% (22/104)	0.0001
CDR per 1000	10.6	48.1	58	0.1249
Cancers diagnosed per 1000 including follow-up	42.6	48.1	58	0.8522
PPV2 (biopsies recommended)	50% (1/2)	25% (5/20)	27.3% (6/22)	0.4805
PPV3 (biopsies performed)	50% (1/2)	25% (5/20)	27.3% (6/22)	0.4805
Sensitivity	25% (1/4)	100% (5/5)	100% (6/6)	0.048
Specificity	96.7% (87/90)	77.8% (77/99)	75.5% (74/98)	0.0001

^a^False negatives includes breast cancers found at follow-up

^b^In the MG plus A-MRI surveillance group, 1 false positive was found with mammography and 2 false positives were found with both MG and A-MRI. All other false positives were found with A-MRI only

^**^p values were obtained comparing the MG group with the MG + A-MRI surveillance groups for intramammary findings only

### Findings according to imaging modality

**Fig. 2:**
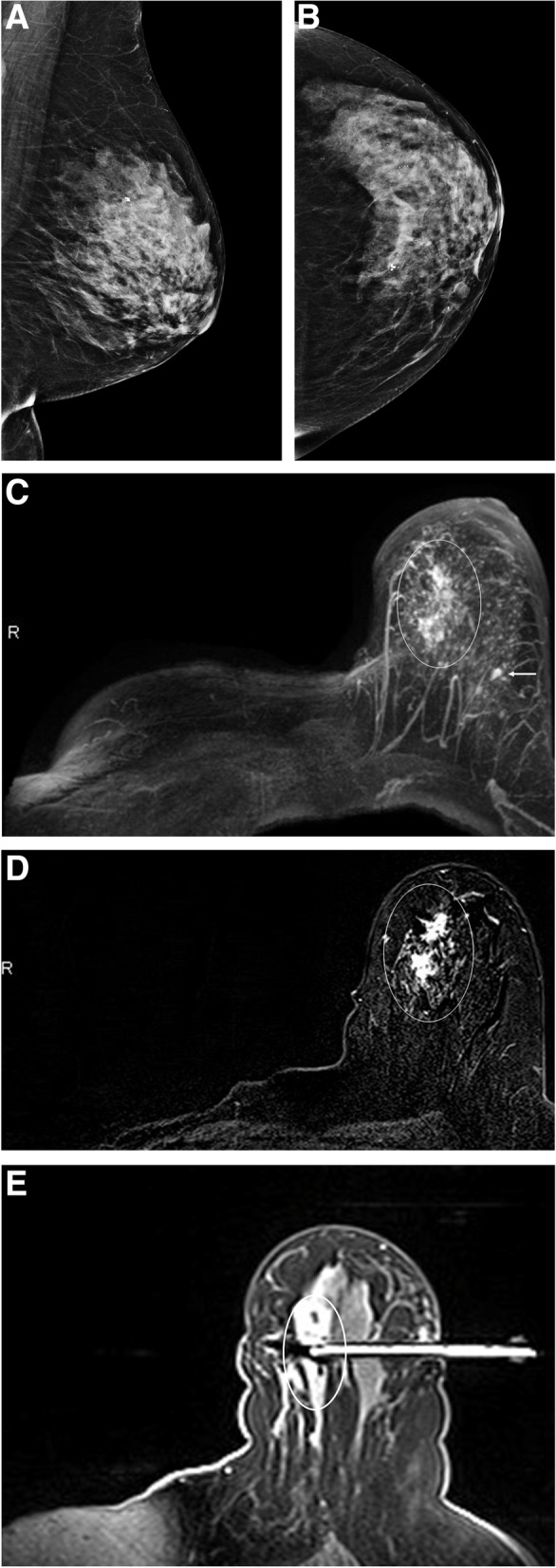
Woman (60–70 years old) with PHBC treated by right mastectomy 12 years prior to study. **A** MLO and **B** CC Left surveillance mammograms show heterogeneously dense breast tissue (BI-RADS C) with normal, stable findings. **C** Axial 3D MIP at 2 min post contrast performed after mammograms shows right mastectomy with no chest wall abnormalities and an irregular 4.5 cm enhancing mass (circle) in the medial left breast reported as BI-RADS 4B. The second circumscribed mass in the lateral breast corresponded with a benign fibroadenoma (arrow). **D** Axial 2 min post contrast subtracted image demonstrates the spiculated enhancing mass in the upper medial quadrant of the left breast (circle). **E** Axial image from MRI biopsy demonstrates the needle sampling the mass (circle), diagnostic for pleomorphic lobular carcinoma in situ, which was confirmed at surgical excision to be associated with invasive lobular carcinoma

Outcomes according to imaging modality are presented in Table 4. Among the 302 imaging examinations performed (198 MG and 104 A-MRI), 9 MG and 29 A-MRI were interpreted as abnormal (17%) (Fig. 2).

**Table 4:** Main imaging findings, BI-RADS category and outcomes according to imaging modality

	**Mammography (*n* = 198)**	**A- MRI (*n* = 104)**	***P* value**
**Normal**	189 (95.5)	75 (72.1)	0.00001
**Recall / abnormal interpretation rate**	9 (4.5%)	29 (27.9)	0.00001
**Mass**	1/9	15/29	
**Calcifications**	6/9	0	
**Asymmetry**	2/9	0	
**Non mass enhancement**	0	9/29	
**Mass/non mass enhancement**	0	1/29	
**Extra-mammary finding**^a^	0	3/29	
**Motion artifact**	0	1/29	
**BI-RADS category after work-up/ full MRI diagnostic protocol**			0.84
**2**	2/9 (22.2%)	2/29 (6.9%)	
**3**	1/9 (11.1%)	4/29 (13.8%)	
**4A**	3/9 (33.3%)	10/29 (34.5%)	
**4B**	3/9 (33.3%)	8/29 (27.6%)	
**5**	0 (0%)	2/29 (6.9%)	
**N/A (extra-mammary findings)**	0 (0%)	3/29 (10.3%)	
**Outcome (abnormal)**			0.49
**Work-up (> benign)**	2/9 (22.2%)	2/29 (6.9%)	
**Stable on follow-up**	1/9 (11.1%)	6/29 (20.7%)	
Biopsied^**^	6/9 (66.7%) (6/198 = 3.0%)	21/29 (72.4%) (21/104 = 20.2%)	
**Outcome of biopsied lesions**			0.99
**Benign**	5/6 (83.3%)	15/21 (71.4%)	
**Atypical/high risk**	0 (0%)	0 (0%)	
**Malignant**	1/6 (16.7%)	6/21(28.6%)	

Unless otherwise indicated, data are numbers of patients and data in parentheses are percentages

^a^Extra-mammary lesions: 2 benign sternal masses (1 biopsied and 1 stable on follow-up) and 1 malignant lung mass (biopsied)

^**^1 suspicious A-MRI finding was less conspicuous the day of the biopsy, for which follow-up was performed and showed stability

### Mammography

There were 198 mammographic examinations performed: 94 MG alone and 104 MG with A-MRI; 95.5% (189/198) were negative or benign (BI-RADS 1 and 2), 4.5% (9/198) were recalled (BI-RADS 0) and 3.0% (6/198) presented findings suspicious for malignancy (BI-RADS 4) and underwent biopsy. One cancer was detected (Table 5) and no high-risk lesions were identified.

**Table 5:** Surveillance detected cancer characteristics

Study ID	Age range	Breast density (ACR)	Months since dx	Group	Abnormality	Detected by	BI-RADS	Histology	TNM	ER	PR	HER 2 neu
1	50 s	C	25	1	Ca +	MG only	4A	DCIS, intermediate nuclear grade Initial ca: IDC, ER-Her +	-			
2	80 s	B	216	2	Mass	A-MRI only	4B	DCIS, high nuclear grade Initial ca: DCIS	-			
3	40 s	C	37	2	Mass	A-MRI only	4B	IDC, grade 2/3 Initial ca: IDC, ER + Her-	T1c N0 M0	+	+	-
4	60 s	C	144	2	NME	A-MRI only	4B	Microinvasive lobular carcinoma, Pleomorphic lobular carcinoma in situ Initial cancer: IDC, TN	T1miN0 M0			
5	30 s	C	7	2	Mass	A-MRI only	5	IDC, grade 3/3 Initial ca: IDC, ER + Her +	T3 N2 M0	-	-	+
6	70 s	C	120	2	Mass	A-MRI only	5	IDC, grade 2/3, DCIS Initial Ca: IDC, TN	T2 Nx	+	-	-
7	50 s	B	33	2	Mass (lung)	A-MRI only	N/A	Adenocarcinoma consistent with breast cancer Initial ca: IDC, ER + Her-	-	+	+	-

Group 1: MG

Group 2: MG plus A-MRI

*DCIS* Ductal carcinoma in situ, *IDC* Invasive ductal carcinoma, *TN* Triple negative, *ER* Estrogen receptor, *Her* Herceptin receptor, *NME* Nonmass enhancement, *NA* Not applicable

**Table 6:** Diagnostic indicators of imaging modalities

	**Mammography**	**A-MRI**^a^	*P* value**
False negative	6	0	
True negative	183	78	
False positive	8	21	
True positive	1	5	
Recall rate	9/198 (4.5%)	25/104 (25%)	< 0.00001
Biopsy rate	6/198 (3.0%)	19/104 (18.3%)	< 0.00001
CDR	1/198 (0.5%)	5/104 (4.8%)	0.0109
PPV2 (recommended)	1/6 (16.7%)	5/20 (25%)	0.59
PPV3 (performed)	1/6 (16.7%)	5/19 (26.3%)	0.557
Sensitivity (%, CI)	1/7 (14.2%, 0.36–57.8)	5/5 (100%)	0.004
Specificity (%, CI)	183/191 (95.8%, 91.9–98)	78/99 (78.8%)	0.00001

*CI* 95% confidence intervals, *A-MRI* Abbreviated breast MRI, *CDR* Cancer detection rate (per 1000 women), *PPV2* Positive predictive value for biopsies recommended, *PPV3* Positive predictive value for biopsies performed, *CI* Confidence interval

^a^including intra-mammary findings only,

^**^excluding extra-mammary findings, chi-square test for comparison between groups

### MRI

104 A-MRI studies were performed; 72.1% (75/104) were negative or benign (BI-RADS 1 and 2); 27.9% (29/104) were abnormal including extra-mammary findings, of which 19.2% (20/104) had suspicious breast lesions (BI-RADS 4 or 5); and 18.2% (19/104) underwent breast biopsy. One breast mass detected by A-MRI was not seen at the time of MRI-guided biopsy and showed stability on 6-month follow-up MRI. Breast cancers were detected in 5 patients; 4 invasive carcinomas and one DCIS (Table 4). All five cancers were only detected with A-MRI, 2 of which were in patients with original triple negative breast cancers (Fig. 2). Three patients had suspicious extra mammary findings: one lung mass seen in the right middle lobe on the A-MRI, and two bone lesions seen in the sternum and manubrium, respectively. The lung mass was confirmed to be a metastatic carcinoma from breast primary on CT guided transthoracic lung biopsy, the manubrial lesion was confirmed to be a hemangioma on bone scan and the sternal lesion was confirmed to be a hibernoma on CT guided biopsy. Of the MRI detected breast cancers, none was identified on mammography, even in retrospect. No high-risk lesions were detected.

The mammographic CDR of 5/1000 (1/98) was significantly lower than the CDR of 58/1000 (6/104, *p* = 0.003) for A-MRI including the extramammary findings, and CDR of 48/1000 (5/104, *p* = 0.0109) for MRI including only the breast findings. The diagnostic indicators for both modalities are presented in Table 6. Sensitivity for MG 14.2% (1/6)) was lower than A-MRI 100% (5/5) (*p* < 0.004); specificity for MG 95.8% (183/191) higher than MRI 76.5% (78/99) (*p* < 0.00001) and PPV3 for MG 16.7% (1/6) was lower than MRI 28.6% (5/19) (*p* = 0.55).

### Necessity for full diagnostic or repeat MRI

Three patients required further investigation requiring diagnostic full MRI based on the radiologist’s uncertainty of the findings seen on the A-MRI: 1 had a mass which was benign on assessment (fat necrosis) determined by the full protocol, one was a BI-RADS 3 lesion which showed stability on 12- month follow-up and one had BI-RADS 4B lesion that led to a benign MRI-guided biopsy of Pseudoangiomatous stromal hyperplasia. A fourth patient had motion artifact and required a repeat abbreviated MRI that was normal and of high technical quality.

### Follow-up

All 191 patients with benign imaging or pathology results underwent clinical and imaging follow-up at the same center, for an average 24 months (10–56 months). There were no cancers found retrospectively as false negatives on follow-up. 1.57% (3/191) had breast cancer on follow-up, all from the surveillance MG only group. Of the 3 cancers diagnosed on *subsequent* surveillance imaging with MG, two were diagnosed at 26 and 27 months with MG (DCIS and invasive ductal carcinoma (IDC), T1N0M0) and one was diagnosed at 50 months on MRI (IDC, T2N0M0). Two were new cancers in the contralateral breast and one DCIS was in the ipsilateral breast; no cancer was seen retrospectively on initial MG and/or MRI. Of the remaining 188 patients with no cancer diagnosed on follow-up, 98.9% (186/188) had follow up of 12 months or longer and 1.06% (2/188) had follow-up of shorter than 12 months. There were no patients lost to follow-up.

## Discussion

This prospective randomized controlled trial showed that undergoing A-MRI had no demonstrable impact on psychological well-being in women with a history of breast cancer. Our primary outcome trait anxiety was moderately high in all patients at baseline and did not change significantly over time; the average score of trait anxiety at the 6-month follow-up was 50.99 ± 4.6 with MG vs 51.73 ± 2.56 with A-MRI, *p* > 0.05. Similarly, self-report worry, including worry of breast cancer recurrence, did not change over time for both conditions. Although there was some benefit of A-MRI over MG alone in reducing state anxiety when participants received their results (T2), which may reflect a higher confidence in women undergoing A-MRI, the difference was less than 4 points and therefore not considered clinically meaningful. Nevertheless, it should be noted that compared to women in the MG alone group, significantly fewer women in the A-MRI group had state anxiety scores in the clinical range. Despite higher rates of biopsies and abnormal interpretations with A-MRI, it is notable that breast A-MRI was not associated with an increase in psychological distress that was sustained over the three time periods. Considering the sustained elevated levels of anxiety and worsening in quality of health in these patients, ongoing consideration and monitoring of mental health issues is recommended. PHBC patients may benefit from psychologist counselling and ongoing support.

Our results support other studies on the impact of breast MRI on anxiety. The Dutch MRI screening (MRISC) study of patients at high risk for breast cancer found that the addition of breast MRI did not affect quality of life or anxiety [20]. In a more recent prospective non-randomized multicentre study, 1561 women at intermediate and high breast cancer risk were noted to have similar moderate distress levels, and there were no more harmful psychological effects observed between standard MG plus ultrasound as compared with the addition of MRI to standard imaging [1].

A significantly higher CDR was noted in the patients who underwent A-MRI as compared with MG only, despite similar baseline demographic and clinical characteristics. We expected a recurrence rate of 1% per year after breast cancer diagnosis [31, 32]. Eighty percent of patients were within 5 years of their breast cancer diagnosis, and 9 in-breast recurrences and 1 lung metastasis were observed, within the expected range. Our study demonstrated A-MRI had a sensitivity of 100% and CDR 48/1000 as compared to mammography’s sensitivity of 14.2% and CDR 5/1000. The low sensitivity of mammography of 14% may be attributed to an early stage of diagnosis of breast cancer in most patients with A-MRI as well as the fact that 49% of women had dense breast tissue, which lowers mammographic sensitivity, with four patients with invasive carcinomas found with A-MRI in women with dense breast tissue (Table 5). The abnormal interpretation and biopsy rates were significantly higher for A-MRI than MG, 25% and 18.3% for A-MRI and 4.5% and 3% for MG, respectively. PPV3 was higher with A-MRI than MG, 26.3% vs 16.7%, although this difference did not reach statistical significance. When extra-mammary findings were included, A-MRI offered the benefit of detecting an incidental lung metastasis.

There have been multiple studies of A-MRI since Dr. Kuhl published her landmark study [13, 15–19, 33–35]. In a similar study of 725 women with PHBC, Choi et al. found 12 cancers using A-MRI, for a CDR 15 per 1000 [34], with comparable sensitivity of 100% and specificity of 89.2%. The results of A-MRI in our study are comparable to reported sensitivities (86–100%) and specificities (45–95.3%). However, our specificity of 76.5% was lower than the ACR benchmark for screening breast MRI 85–90% [22]. PPV3 26.3% was in the reported range for A-MRI (9.2–70.2%) and met the ACR benchmark of 20–50% [22]. In 2020, Park et al. retrospectively compared abbreviated to full MRI in 1200 women with PHBC, 656 with A-MRI vs 656 patients with full protocol and found no significant differences in sensitivity (70% vs 100%) or specificity (98% vs 96.9%), negative predictive values (99.5 and 100%) and PPV (35% vs 23%) (all *p* > 0.05) [36].

We recognize some limitations of our study. Patients were recruited by their oncologists or treating surgeons, which could have introduced a bias in patient selection. This may have partly explained the CDR in the MG plus A-MRI group. Nonetheless, the fact that randomization was blinded mitigated any potential bias of intervention arm selection and there were no clinical differences between the two surveillance groups. As well, in follow-up, similar numbers of cancers were detected in each surveillance group. This may indicate earlier detection of cancers with A-MRI than interval cancers in the MG group. Because of the CDRs and minimal effect on anxiety in the MRI group we stopped the clinical trial early. Additionally, some patients could have developed breast cancer after the follow up period, which might have been missed with mammography. Given that the majority of patients were followed for over 24 months, this is less likely. Another limitation is that the radiologists were not blinded to the allocation arm, which could have influenced their reporting of the mammogram, if they knew that an MRI would be done. However, given similar recall rates for mammography within both groups, this is unlikely to have been present. The high biopsy rate in the MRI group may be perceived as a limitation, but this was related to the high CDR with an acceptable PPV3. In this study, all additional biopsies and follow-up imaging were fully covered by the publicly funded health care system, as per standard of care. However, more research is required to find ways to further reduce the rate of false positives. There is likely a learning curve with A-MRI and the addition of T2 sequences may help to improve PPV3 without significant time cost [16]. We have subsequently adapted an abbreviated protocol to include T2 sequence and two more post contrast sequences to improve the specificity of MRI [37]. This was a short-term trial and did not account for long-term follow-up for anxiety measures and other clinical outcomes with repeated breast imaging. For example, the false positive rate is often higher for the baseline screening breast MRI and specificity tends to increase with repeated breast imaging with a baseline for comparison. Another limitation is that assessment of anxiety was based on self-report questionnaires and limited by the time points in which it is measured. The study was powered to 134 patients per group to measure a clinically meaningful difference in anxiety and it is possible that the study was underpowered. There was also a loss of data of the primary outcome for participants over time, which could have led to underestimation of the effect of the surveillance group on levels of anxiety. A more objective measure would be to evaluate adherence to follow-up rounds of screening, which may address poor compliance with MRI screening [14]. This is recommended for future study. Also, our study lacked the sample size and enough long-term follow-up to be able to say whether the earlier detection in the A-MRI group led to any difference in survival. This trial was conducted at a single institution, tertiary care academic center with 2 breast radiologists reviewing all the breast imaging. Therefore, these trial results may not be generalizable to other clinical settings. The imaging surveillance tests in the study were usually done on the same day although in some (17% (18/104)), the studies were performed on different days with a large interval time between MG and A-MRI (average 33.2 days (range: 1–147). Among women at high-risk for breast cancer, annual mammograms and breast MRIs are often staggered every 6 months to try to reduce the incidence of interval cancers and would reflect clinical differences from our study.

In conclusion, the addition of A- MRI to surveillance mammography did not impact patient anxiety in women with PHBC, regardless of the significantly higher recall and biopsy rates. A-MRI showed significantly higher cancer detection rate compared to mammography alone, which is consistent with recent recommendations. Although further study with larger cohorts is warranted, an abbreviated protocol may be considered for surveillance in this population.

## Supplementary Information

**Additional file 1.** Questionnaires used in the study. Description of the questionnaires used in the study.

## Data Availability

The data will be deposited in the uOttawa—Dataverse (Université d’Ottawa / University of Ottawa): https://borealisdata.ca/ and data are available upon request.

## Abbreviations

A-MRI: Abbreviated breast magnetic resonance imaging
MG: Mammography
CDR: Cancer detection rate
DCIS: Ductal carcinoma in situ
IDC: Invasive ductal carcinoma
PHBC: Prior (personal) history of breast cancer
BI-RADS: Breast Imaging-Reporting Data System
CDR: Cancer detection rate
AIR: Abnormal interpretation rate
PPV: Positive predictive value
