# Correction: Randomized trial of surveillance with abbreviated MRI in women with a personal history of breast cancer– impact on patient anxiety and cancer detection

**DOI:** 10.1186/s12885-022-10108-2

**Published:** 2022-09-23

**Authors:** Marina Mohallem Fonseca, Tasneem Alhassan, Yashmin Nisha, Diana Koszycki, Betty Anne Schwarz, Roanne Segal, Angel Arnaout, Tim Ramsay, Jacqueline Lau, Jean M. Seely

**Affiliations:** 1 Breast Imaging fellow 2017-2018, Former University of Ottawa, Now Annecy, France; 2 Breast Imaging fellow 2016-2017, Former University of Ottawa, Now Dubai, United Arab Emirates; 3 University of Ottawa, Breast Imaging fellow, Ottawa, 2019-2020 Canada; 4 Research Chair in Mental Health, Institut du Savoir Montfort, Ottawa, Canada; 5 Faculty of Education (Counselling Psychology), Faculty of Medicine (Psychiatry), Institut du Savoir Monfort, Ottawa, Canada; 6 Department of Medical Imaging, The Ottawa Hospital, Ottawa, Canada; 7 Department of Medicine, Oncology, The Ottawa Hospital Cancer Center, University of Ottawa, Ottawa, Canada; 8 Breast Surgical Oncology and Oncoplastic Surgery, University of Ottawa, Ottawa Hospital Research Institute, Ottawa, Canada; 9 Clinical Epidemiology Program, School of Epidemiology and Public Health, Ottawa Hospital Research Institute, University of Ottawa, Ottawa, Canada; 10 Department of Radiology, University of Ottawa, Ottawa, Canada; 11 Departments of Radiology and Surgery, Department of Medical Imaging, The Ottawa Hospital, Ottawa Hospital Research Institute, University of Ottawa, General Campus, 501 Smyth Rd, Ottawa, ON K1H 8L6 Canada

**Correction: BMC Cancer 22, 774 (2022)**

**https://doi.org/10.1186/s12885-022-09792-x**

Following publication of the original article [1], the authors identified an error in the author name of Tasneem Alhassan.

The incorrect author name is: Tasneen Alhassan

The correct author name is: Tasneem Alhassan

The author group has been updated above and the original article [1] has been corrected.
