# Supplementary material for: Randomized trial of surveillance with abbreviated MRI in women with a personal history of breast cancer– impact on patient anxiety and cancer detection
Source: BMC Cancer. 2022 Jul 15;22:774. doi: 10.1186/s12885-022-09792-x (PMC9287889; doi:10.1186/s12885-022-09792-x)
Supplement: Supplementary file 1 — Additional file 1. Questionnaires used in the study. Description of the questionnaires used in the study. [file 12885_2022_9792_MOESM1_ESM.pdf]

Breast Cancer Worry Questionnaire

Study ID:_________________________ Date: __________________

Please circle the response which best describes your feelings.

Worry Frequency

1. How worried are you about getting breast cancer someday?

1 = not at all; 2 = rarely; 3 = sometimes; 4 = often; 5 = almost all the time

Worry Impact

2. How much does your worry affect your mood?

1 = not at all; 2 = a little; 3 = somewhat; 4 = a lot

3. How much does your worry affect your ability to perform your daily activities?

1 = not at all; 2 = a little; 3 = somewhat; 4 = a lot

Patient ID#: ______________ Date: _____________

Health Status Questionnaire (HSQ-12)

1. In general, would you say your health is: (Circle one number)

Excellent………1
Very Good…….2
Good… 3
Fair…...............4
Poor…..............5

The following items are about activities you might do during a typical day. Does your health now limit you in these activities? If so, how much? (Circle one number on each line)

	Yes, Limited a lot	Yes, limited a little	No, not limited at all
2. Lifting or carrying groceries	1	2	3
3. Climbing several flights of stairs	1	2	3
4. Walking several blocks	1	2	3

5. During the past 4 weeks how much difficulty did you have doing your work or other regular daily activities as a result of your physical health? (Circle one number)

None at all…............... 1
A little bit….................2
Moderately…................3
Quite a bit… 4
Couldn't do any work…5

6. During the past 4 weeks, to what extent have you accomplished less than you would like in your work or other daily activities as a result of emotional problems (such as feeling depressed or anxious)? (Circle one number)

None at all….................1
A little bit….................2
Moderately…................3
Quite a bit… 4
Couldn't do any work…5

7. During the past 4 weeks, to what extent has your physical health or emotional problems interfered with your normal social activities with family, friends, neighbours, or groups? (Circle one number)

None at all…............... 1
A little bit….................2
Moderately…...............3
Quite a bit… 4
Couldn't do any work…5

8. How much bodily pain have you had during the past 4 weeks? (Circle one number)

None…......................... 1
Very mild…................ 2
Mild 3
Moderate…................... 4
Severe 5
Very severe….............. 6

These questions are about how you feel things have been with you during the past 4 weeks. For each question, please give the one answer that comes closest to the way you have been feeling.

How much of the time during the past 4 weeks… (Circle one number on each line)

	All of the time	Most of the time	A good bit of the time	Some of the time	Little of the time	None of the time
9. Have you felt calm and peaceful?	1	2	3	4	5	6
10. Did you have a lot of energy?	1	2	3	4	5	6
11. Have you felt downhearted and blue?	1	2	3	4	5	6
12. Have you been happy?	1	2	3	4	5	6

Patient ID:______________________________________ Date:_______________

The Penn State Worry Questionnaire (PSWQ)

Instructions: Rate each of the following statements on a scale of 1 (“not at all typical of me”) to 5 (“very typical of me”). Please do not leave any items blank.

	Not at all typical of me				Very typical of me
1. If I do not have enough time to do everything, I do not worry about it.	1	2	3	4	5
2. My worries overwhelm me.	1	2	3	4	5
3. I do not tend to worry about things.	1	2	3	4	5
4. Many situations make me worry.	1	2	3	4	5
5. I know I should not worry about things, but I just cannot help it.	1	2	3	4	5
6. When I am under pressure I worry a lot.	1	2	3	4	5
7. I am always worrying about something.	1	2	3	4	5
8. I find it easy to dismiss worrisome thoughts.	1	2	3	4	5
9. As soon as I finish one task, I start to worry about everything else I have to do.	1	2	3	4	5
10. I never worry about anything.	1	2	3	4	5
11. When there is nothing more I can do about a concern, I do not worry about it any more.	1	2	3	4	5
12. I have been a worrier all my life.	1	2	3	4	5
13. I notice that I have been worrying about things.	1	2	3	4	5
14. Once I start worrying, I cannot stop.	1	2	3	4	5
15. I worry all the time.	1	2	3	4	5
16. I worry about projects until they are all done.	1	2	3	4	5
